# Supplementary material for: A Likelihood Approach for Real-Time Calibration of Stochastic Compartmental Epidemic Models
Source: PLoS Comput Biol. 2017 Jan 17;13(1):e1005257. doi: 10.1371/journal.pcbi.1005257 (PMC5240920; doi:10.1371/journal.pcbi.1005257)
Supplement: S1 File — (TAR.GZ) [file pcbi.1005257.s014.tar.gz › HSPH_Online-SI-Revision/PF08/n100-severe/n100-severe_PF2_table-4.pdf]

1	newly 3 weeks spec	6483.	5559.73	{5124., 5916.}	14.2414	0.0673772	newly 3 weeks cumul	16417.	14149.4	{13063., 15107.}	13.8125	0.0651633
2	newly 3 weeks spec	9252.	8564.52	{8268., 8914.}	7.43061	0.0337711	newly 3 weeks cumul	20576.	18524.9	{17601., 19555.}	9.96851	0.0459664
3	newly 3 weeks spec	13618.	7998.07	{7603., 8606.}	41.2684	0.231654	newly 3 weeks cumul	28056.	16846.6	{16039., 18018.}	39.9537	0.222047
4	newly 3 weeks spec	8496.	8725.46	{8084., 9360.}	4.82086	0.0203779	newly 3 weeks cumul	19445.	19639.8	{18095., 21067.}	4.60483	0.0197582
5	newly 3 weeks spec	5838.	6270.19	{5697., 6862.}	8.25403	0.0338317	newly 3 weeks cumul	15689.	16387.3	{14866., 17937.}	6.53579	0.0273394
6	newly 3 weeks spec	8007.	6645.02	{6207., 7041.}	17.0099	0.0814764	newly 3 weeks cumul	17018.	14427.6	{13584., 15396.}	15.2218	0.0722605
7	newly 3 weeks spec	9834.	7541.02	{6482., 8111.}	23.3169	0.11681	newly 3 weeks cumul	19745.	15796.3	{13900., 17091.}	19.9987	0.0985744
8	newly 3 weeks spec	6637.	5769.64	{5320., 6321.}	13.0686	0.0615951	newly 3 weeks cumul	16097.	13679.3	{12766., 14859.}	15.0193	0.0713308
9	newly 3 weeks spec	10162.	2253.63	{1549., 2712.}	77.823	0.665564	newly 3 weeks cumul	16274.	4551.11	{3218., 5458.}	72.0345	0.562608
10	newly 3 weeks spec	8608.	3216.14	{3113., 3331.}	62.6378	0.427741	newly 3 weeks cumul	19464.	7807.08	{7633., 7951.}	59.8896	0.396827
11	newly 3 weeks spec	9885.	7739.3	{7488., 8003.}	21.7066	0.106414	newly 3 weeks cumul	20715.	16190.1	{15649., 16778.}	21.8438	0.107227
12	newly 3 weeks spec	6820.	4091.76	{3943., 4211.}	40.0035	0.222021	newly 3 weeks cumul	17074.	10184.	{9917., 10414.}	40.3539	0.22449
13	newly 3 weeks spec	6696.	5804.08	{5420., 6116.}	13.3202	0.0625801	newly 3 weeks cumul	17080.	14947.3	{13978., 15781.}	12.4865	0.0583838
14	newly 3 weeks spec	5021.	5092.21	{4763., 5380.}	4.34196	0.0188408	newly 3 weeks cumul	12730.	12750.3	{11898., 13437.}	4.3176	0.0189915
15	newly 3 weeks spec	6167.	4617.14	{4269., 4938.}	25.1315	0.126459	newly 3 weeks cumul	14161.	10977.	{10261., 11672.}	22.4846	0.111247
16	newly 3 weeks spec	5400.	4320.97	{3976., 4827.}	19.982	0.0980099	newly 3 weeks cumul	13040.	10554.6	{9768., 11681.}	19.06	0.0928686
17	newly 3 weeks spec	7444.	5733.54	{4476., 6445.}	22.9777	0.11706	newly 3 weeks cumul	14892.	11716.6	{9422., 13039.}	21.3229	0.107254
18	newly 3 weeks spec	7109.	7287.29	{6470., 8177.}	8.15797	0.0343042	newly 3 weeks cumul	17024.	16544.8	{14648., 18371.}	7.81761	0.034657
19	newly 3 weeks spec	9821.	8975.28	{8504., 9311.}	8.61134	0.039362	newly 3 weeks cumul	23696.	21077.	{20116., 21909.}	11.0525	0.0511068
20	newly 3 weeks spec	8828.	5241.38	{5057., 5411.}	40.6278	0.226674	newly 3 weeks cumul	20393.	12226.9	{11850., 12544.}	40.0437	0.222409
21	newly 3 weeks spec	9444.	7193.84	{6350., 8005.}	23.8263	0.119715	newly 3 weeks cumul	18625.	14932.3	{13126., 16871.}	19.8264	0.0977174
22	newly 3 weeks spec	7683.	3360.08	{3049., 3627.}	56.266	0.36103	newly 3 weeks cumul	14543.	7370.34	{6655., 7873.}	49.3204	0.296711
23	newly 3 weeks spec	16661.	4761.16	{3364., 5474.}	71.4233	0.549681	newly 3 weeks cumul	28675.	9260.33	{6886., 10586.}	67.7059	0.495775
24	newly 3 weeks spec	11463.	7069.85	{3805., 9221.}	38.3246	0.23689	newly 3 weeks cumul	20345.	12819.8	{7539., 16667.}	36.988	0.222749
25	newly 3 weeks spec	11140.	6912.58	{4218., 9582.}	37.9481	0.233927	newly 3 weeks cumul	19297.	12542.2	{8070., 17230.}	35.0047	0.209068
26	newly 3 weeks spec	9973.	4741.83	{4590., 4893.}	52.4533	0.322999	newly 3 weeks cumul	21090.	10758.8	{10574., 10956.}	48.9865	0.29237
27	newly 3 weeks spec	9435.	7142.78	{6939., 7329.}	24.2949	0.120955	newly 3 weeks cumul	19821.	15712.8	{15429., 15971.}	20.7267	0.100909
28	newly 3 weeks spec	6339.	7405.1	{6809., 7894.}	16.9206	0.0670959	newly 3 weeks cumul	16315.	18114.1	{16700., 19194.}	11.4145	0.046299
29	newly 3 weeks spec	9036.	9168.75	{8776., 9547.}	2.8195	0.012084	newly 3 weeks cumul	21261.	20966.5	{20135., 21820.}	2.87517	0.0126554
30	newly 3 weeks spec	6346.	4226.81	{4079., 4355.}	33.3941	0.176651	newly 3 weeks cumul	15783.	10362.3	{10132., 10583.}	34.3452	0.182853
31	newly 3 weeks spec	15806.	6335.31	{3074., 8931.}	59.9183	0.432496	newly 3 weeks cumul	27617.	11489.1	{6077., 15642.}	58.3984	0.40869
32	newly 3 weeks spec	10271.	5420.97	{3723., 6245.}	47.2206	0.283836	newly 3 weeks cumul	19060.	10712.2	{7744., 12313.}	43.7976	0.255473
33	newly 3 weeks spec	12313.	8371.4	{8050., 8588.}	32.0117	0.168145	newly 3 weeks cumul	26701.	17790.9	{17114., 18143.}	33.3698	0.176931
34	newly 3 weeks spec	7987.	3173.35	{3100., 3274.}	60.2686	0.400975	newly 3 weeks cumul	19140.	7773.84	{7645., 7891.}	59.3843	0.391344
35	newly 3 weeks spec	12123.	3848.7	{2669., 4762.}	68.2529	0.513443	newly 3 weeks cumul	20957.	7481.58	{5481., 9120.}	64.3003	0.45955
36	newly 3 weeks spec	8896.	4977.53	{3893., 5524.}	44.0475	0.255959	newly 3 weeks cumul	16239.	9943.73	{8157., 10985.}	38.7664	0.216203
37	newly 3 weeks spec	11908.	9665.79	{6258., 10553.}	18.8294	0.0972174	newly 3 weeks cumul	22407.	18349.7	{12633., 19828.}	18.1072	0.0925109
38	newly 3 weeks spec	11993.	7652.49	{7406., 7879.}	36.192	0.195242	newly 3 weeks cumul	24507.	16031.1	{15488., 16439.}	34.5857	0.184487
39	newly 3 weeks spec	6786.	7349.	{6620., 7790.}	9.3354	0.0384741	newly 3 weeks cumul	16864.	17683.6	{16062., 18839.}	6.81179	0.0286409
40	newly 3 weeks spec	7110.	8186.42	{6729., 8910.}	17.5823	0.070063	newly 3 weeks cumul	16261.	17591.5	{14603., 19414.}	12.1432	0.0504252
41	newly 3 weeks spec	12763.	11069.9	{10553., 11727.}	13.2658	0.0627817	newly 3 weeks cumul	25803.	21987.9	{21012., 23149.}	14.7856	0.0704235
42	newly 3 weeks spec	7600.	4870.6	{4412., 5105.}	35.9132	0.193921	newly 3 weeks cumul	17610.	11603.9	{10615., 12052.}	34.1062	0.181689
43	newly 3 weeks spec	5514.	5166.25	{4621., 5583.}	7.03464	0.0325929	newly 3 weeks cumul	14068.	12682.1	{11491., 13544.}	9.88655	0.0462847
44	newly 3 weeks spec	9897.	5497.51	{5073., 6160.}	44.4528	0.259202	newly 3 weeks cumul	17922.	10831.4	{10027., 12257.}	39.5634	0.222072
45	newly 3 weeks spec	7988.	6542.08	{6020., 6992.}	18.1012	0.0873573	newly 3 weeks cumul	17198.	14092.5	{13009., 15038.}	18.0575	0.0870898
46	newly 3 weeks spec	8753.	3597.46	{3363., 3788.}	58.9003	0.386605	newly 3 weeks cumul	17795.	7927.21	{7449., 8284.}	55.4526	0.351569
47	newly 3 weeks spec	6293.	6533.14	{6096., 6907.}	5.04147	0.0212348	newly 3 weeks cumul	16455.	16785.4	{15649., 17738.}	4.30763	0.0184018
48	newly 3 weeks spec	8060.	8241.72	{7568., 8747.}	5.01762	0.0214696	newly 3 weeks cumul	18107.	18329.6	{16819., 19336.}	4.81659	0.0207567
49	newly 3 weeks spec	13841.	3727.31	{1767., 7073.}	73.0705	0.625575	newly 3 weeks cumul	23044.	6847.88	{3538., 12369.}	70.2835	0.571423
50	newly 3 weeks spec	12684.	10767.5	{10183., 11438.}	15.1093	0.0727775	newly 3 weeks cumul	25589.	21234.9	{20177., 22698.}	17.0157	0.0824852

1	newly 5 weeks spec	6768.	6408.54	{5968., 6832.}	5.88357	0.0267248	newly 5 weeks cumul	30019.	26718.	{24826., 28447.}	10.9965	0.0511766
2	newly 5 weeks spec	7700.	10776.6	{10611., 10923.}	39.9564	0.145946	newly 5 weeks cumul	37668.	39585.7	{38428., 40693.}	5.26622	0.0222274
3	newly 5 weeks spec	10200.	10755.9	{10438., 11154.}	5.7448	0.0241733	newly 5 weeks cumul	51910.	37581.4	{36341., 39598.}	27.6027	0.140612
4	newly 5 weeks spec	8732.	10297.1	{9915., 10692.}	17.9236	0.0714437	newly 5 weeks cumul	37352.	40001.1	{37514., 42417.}	7.19008	0.0297556
5	newly 5 weeks spec	5980.	6698.27	{6191., 7298.}	12.0112	0.0485073	newly 5 weeks cumul	27938.	29748.8	{27155., 32462.}	7.40189	0.0305348
6	newly 5 weeks spec	9118.	9264.56	{8815., 9644.}	2.90349	0.0124577	newly 5 weeks cumul	35492.	31998.3	{30235., 33623.}	9.84374	0.0453369
7	newly 5 weeks spec	11408.	10516.1	{9565., 10887.}	7.81864	0.0358302	newly 5 weeks cumul	43078.	35861.1	{31706., 37940.}	16.7532	0.0806231
8	newly 5 weeks spec	7091.	7426.3	{6953., 7902.}	5.42942	0.0226995	newly 5 weeks cumul	30302.	27893.7	{26105., 30103.}	8.04148	0.036921
9	newly 5 weeks spec	13814.	4817.4	{2985., 5795.}	65.1267	0.4732	newly 5 weeks cumul	44791.	12767.8	{8621., 15368.}	71.4947	0.557526
10	newly 5 weeks spec	8668.	4473.84	{4315., 4602.}	48.3867	0.287374	newly 5 weeks cumul	37387.	16154.3	{15831., 16442.}	56.7917	0.364506
11	newly 5 weeks spec	9915.	10706.5	{10550., 10877.}	7.98245	0.0333201	newly 5 weeks cumul	41751.	36688.1	{36005., 37557.}	12.1264	0.0562072
12	newly 5 weeks spec	6925.	5236.94	{5076., 5369.}	24.3763	0.121429	newly 5 weeks cumul	31324.	20162.6	{19723., 20563.}	35.632	0.19139
13	newly 5 weeks spec	6771.	6459.73	{6076., 6833.}	5.20854	0.0234405	newly 5 weeks cumul	30784.	27689.	{25924., 29099.}	10.0539	0.0464306
14	newly 5 weeks spec	5391.	6132.92	{5878., 6382.}	14.023	0.0567629	newly 5 weeks cumul	23614.	24624.7	{23330., 25768.}	5.58584	0.0236965
15	newly 5 weeks spec	6966.	6290.08	{5826., 6680.}	9.70313	0.0448891	newly 5 weeks cumul	28290.	22833.8	{21195., 24309.}	19.2868	0.0936454
16	newly 5 weeks spec	6295.	5630.39	{5172., 6285.}	10.7782	0.0504823	newly 5 weeks cumul	25735.	21246.	{19730., 23573.}	17.443	0.0842628
17	newly 5 weeks spec	8949.	9342.53	{7419., 10117.}	9.6061	0.0415589	newly 5 weeks cumul	32852.	28861.3	{22859., 31903.}	12.4344	0.0603701
18	newly 5 weeks spec	7086.	9116.13	{8353., 9806.}	28.6499	0.108379	newly 5 weeks cumul	31968.	34263.5	{30773., 37837.}	8.86421	0.0360329
19	newly 5 weeks spec	7954.	9864.83	{9527., 10107.}	24.0235	0.0933937	newly 5 weeks cumul	41504.	40872.2	{39289., 42176.}	2.361	0.0104338
20	newly 5 weeks spec	8356.	7140.19	{6986., 7313.}	14.5501	0.068452	newly 5 weeks cumul	37963.	25724.1	{25017., 26335.}	32.239	0.169213
21	newly 5 weeks spec	10527.	10381.4	{9606., 10803.}	3.36991	0.0150586	newly 5 weeks cumul	40402.	34586.4	{31235., 37415.}	14.3945	0.0685236
22	newly 5 weeks spec	9422.	5630.54	{5220., 6071.}	40.2405	0.225589	newly 5 weeks cumul	33493.	17489.1	{15972., 18870.}	47.7828	0.283911
23	newly 5 weeks spec	10882.	8970.5	{6806., 9998.}	17.5657	0.0878214	newly 5 weeks cumul	56693.	25248.7	{18609., 28637.}	55.4642	0.355817
24	newly 5 weeks spec	12907.	12133.	{7994., 14376.}	18.2416	0.0930947	newly 5 weeks cumul	47801.	35316.8	{21259., 44133.}	26.1171	0.151554
25	newly 5 weeks spec	11133.	12004.3	{8859., 14531.}	25.2043	0.107445	newly 5 weeks cumul	44204.	34737.2	{23720., 44809.}	22.349	0.127374
26	newly 5 weeks spec	10139.	6924.78	{6729., 7113.}	31.7015	0.165683	newly 5 weeks cumul	42295.	23628.2	{23255., 24061.}	44.1348	0.252904
27	newly 5 weeks spec	9500.	9587.35	{9406., 9738.}	1.33379	0.00574914	newly 5 weeks cumul	40049.	34056.5	{33488., 34523.}	14.9629	0.0704173
28	newly 5 weeks spec	5894.	8216.88	{7680., 8694.}	39.4109	0.143746	newly 5 weeks cumul	28643.	34468.1	{32424., 36377.}	20.3369	0.0797242
29	newly 5 weeks spec	8506.	10368.3	{10056., 10600.}	21.8938	0.0858936	newly 5 weeks cumul	39366.	41697.3	{40227., 42905.}	5.94996	0.0249598
30	newly 5 weeks spec	6273.	5469.28	{5271., 5616.}	12.8124	0.0596846	newly 5 weeks cumul	28864.	20768.3	{20265., 21206.}	28.0478	0.143067
31	newly 5 weeks spec	9080.	11366.8	{6257., 14568.}	39.1597	0.146771	newly 5 weeks cumul	52596.	32301.9	{16748., 43047.}	38.5849	0.238925
32	newly 5 weeks spec	11191.	9491.39	{7254., 10322.}	15.1873	0.0760958	newly 5 weeks cumul	42799.	27885.2	{20671., 31161.}	34.846	0.191073
33	newly 5 weeks spec	10094.	10905.1	{10691., 11181.}	8.39667	0.0349294	newly 5 weeks cumul	49375.	38961.6	{37962., 39617.}	21.0904	0.103252
34	newly 5 weeks spec	7767.	4333.68	{4202., 4465.}	44.2039	0.253509	newly 5 weeks cumul	35440.	15891.3	{15579., 16217.}	55.1599	0.348381
35	newly 5 weeks spec	11360.	7739.81	{5323., 9340.}	31.8679	0.181098	newly 5 weeks cumul	47195.	21026.8	{14787., 25785.}	55.447	0.365112
36	newly 5 weeks spec	12338.	8898.15	{6901., 9695.}	27.8801	0.144952	newly 5 weeks cumul	40963.	25939.6	{20425., 28446.}	36.6755	0.201652
37	newly 5 weeks spec	9251.	13268.7	{9942., 13874.}	43.5048	0.154374	newly 5 weeks cumul	44615.	44276.3	{30873., 47052.}	7.12267	0.0346907
38	newly 5 weeks spec	11210.	10596.4	{10422., 10724.}	5.47386	0.0244756	newly 5 weeks cumul	48530.	36296.9	{35559., 37038.}	25.2074	0.126201
39	newly 5 weeks spec	6041.	8470.58	{7883., 8893.}	40.2182	0.146341	newly 5 weeks cumul	29945.	34404.	{31304., 36258.}	14.9006	0.0597322
40	newly 5 weeks spec	7170.	10604.1	{9329., 11071.}	47.896	0.168991	newly 5 weeks cumul	31380.	38155.9	{32444., 40919.}	22.0156	0.0851894
41	newly 5 weeks spec	9905.	13312.2	{13051., 13699.}	34.3987	0.128146	newly 5 weeks cumul	48570.	48752.2	{47305., 50803.}	2.73646	0.0121989
42	newly 5 weeks spec	7814.	6537.86	{5983., 6842.}	16.3315	0.0780208	newly 5 weeks cumul	33674.	23980.3	{21909., 24948.}	28.7868	0.148
43	newly 5 weeks spec	5382.	6455.07	{5816., 6921.}	19.9381	0.0779227	newly 5 weeks cumul	25003.	25076.4	{22666., 26757.}	6.15482	0.0269635
44	newly 5 weeks spec	12427.	9678.53	{9392., 10240.}	22.1169	0.111193	newly 5 weeks cumul	43675.	28370.9	{26713., 31031.}	35.0408	0.190398
45	newly 5 weeks spec	9497.	9323.73	{8847., 9762.}	3.35506	0.0148871	newly 5 weeks cumul	36455.	31697.9	{29506., 33376.}	13.0493	0.0611441
46	newly 5 weeks spec	10647.	5898.98	{5565., 6189.}	44.5949	0.256879	newly 5 weeks cumul	39229.	18593.4	{17541., 19548.}	52.603	0.32463
47	newly 5 weeks spec	6515.	7179.98	{6857., 7484.}	10.2069	0.0419786	newly 5 weeks cumul	29490.	31024.9	{29388., 32517.}	5.55449	0.0232375
48	newly 5 weeks spec	8793.	10133.5	{9693., 10495.}	15.264	0.061471	newly 5 weeks cumul	35919.	38167.5	{35435., 39886.}	7.13227	0.0297534
49	newly 5 weeks spec	12770.	7913.49	{3803., 13687.}	41.3684	0.266622	newly 5 weeks cumul	52454.	20641.7	{10075., 37188.}	60.6481	0.453026
50	newly 5 weeks spec	9733.	13339.6	{13073., 13705.}	37.055	0.136475	newly 5 weeks cumul	48457.	47863.8	{46295., 49931.}	3.28295	0.0151285

1	newly 54 weeks cumul	56585.	73779.1	{71403., 76848.}	30.3864	0.115008
2	newly 61 weeks cumul	50118.	91186.2	{90781., 91588.}	81.943	0.259928
3	newly 62 weeks cumul	64948.	90969.5	{91079., 92060.}	40.0652	0.146177
4	newly 60 weeks cumul	61359.	89188.8	{88439., 90062.}	45.3557	0.162423
5	newly 54 weeks cumul	56535.	72809.6	{70062., 76828.}	28.7868	0.109541
6	newly 60 weeks cumul	63071.	88369.3	{87718., 89001.}	40.1108	0.146465
7	newly 61 weeks cumul	66246.	91443.6	{90528., 92365.}	38.0364	0.139934
8	newly 57 weeks cumul	53290.	79429.4	{78385., 80265.}	49.0512	0.173315
9	newly 62 weeks cumul	61547.	85676.1	{65622., 90171.}	39.72	0.143308
10	newly 55 weeks cumul	57915.	75027.9	{74548., 75539.}	29.5483	0.112427
11	newly 61 weeks cumul	59863.	91840.8	{91615., 92049.}	53.4184	0.185877
12	newly 54 weeks cumul	59414.	74032.6	{73554., 74454.}	24.6047	0.0955301
13	newly 54 weeks cumul	57756.	72694.	{70455., 76125.}	25.864	0.0997133
14	newly 54 weeks cumul	54929.	73915.7	{71670., 78424.}	34.5658	0.128673
15	newly 56 weeks cumul	48068.	80288.6	{75512., 81564.}	67.0312	0.222676
16	newly 55 weeks cumul	58209.	74538.1	{72391., 78845.}	28.0525	0.107127
17	newly 61 weeks cumul	50701.	90493.7	{88723., 92492.}	78.4851	0.251158
18	newly 59 weeks cumul	50495.	82625.6	{74456., 88381.}	63.6313	0.212617
19	newly 59 weeks cumul	56218.	87012.4	{86000., 87854.}	54.7767	0.189689
20	newly 57 weeks cumul	56638.	83288.8	{82946., 83668.}	47.0546	0.16747
21	newly 61 weeks cumul	60507.	91629.4	{90810., 92375.}	51.436	0.18021
22	newly 59 weeks cumul	53168.	80345.8	{78941., 81564.}	51.1169	0.179092
23	newly 63 weeks cumul	64940.	88837.2	{81230., 92849.}	36.7988	0.135275
24	newly 64 weeks cumul	66552.	92286.3	{90052., 93919.}	38.9623	0.142593
25	newly 64 weeks cumul	57475.	92989.1	{90611., 93964.}	61.7905	0.208863
26	newly 58 weeks cumul	65347.	81368.6	{81104., 81667.}	24.5177	0.0952289
27	newly 59 weeks cumul	58495.	89113.5	{88895., 89325.}	52.3438	0.182824
28	newly 58 weeks cumul	47081.	76032.7	{67562., 82989.}	61.4933	0.206335
29	newly 59 weeks cumul	60373.	89105.6	{88164., 89907.}	47.5919	0.169051
30	newly 55 weeks cumul	48852.	72839.8	{72383., 73263.}	49.1029	0.173481
31	newly 64 weeks cumul	59587.	91839.2	{86833., 94065.}	54.1842	0.187494
32	newly 62 weeks cumul	60379.	91764.8	{86798., 93191.}	51.9814	0.181527
33	newly 62 weeks cumul	64967.	91785.	{91803., 93088.}	41.2794	0.14992
34	newly 54 weeks cumul	57950.	73629.7	{73116., 74052.}	27.0573	0.103994
35	newly 63 weeks cumul	60555.	88076.6	{81343., 92955.}	45.5758	0.161978
36	newly 61 weeks cumul	64577.	90643.	{89497., 93069.}	40.3642	0.146485
37	newly 63 weeks cumul	54448.	91461.7	{91704., 92483.}	67.9799	0.225022
38	newly 61 weeks cumul	66756.	91305.1	{91080., 91522.}	36.7743	0.136004
39	newly 58 weeks cumul	46740.	77025.8	{72691., 84689.}	64.7963	0.216257
40	newly 61 weeks cumul	47800.	90591.8	{86517., 92591.}	89.5227	0.277197
41	newly 63 weeks cumul	62184.	93334.9	{92851., 93733.}	50.0947	0.176363
42	newly 56 weeks cumul	56523.	80939.3	{75852., 82230.}	43.1971	0.155793
43	newly 55 weeks cumul	42797.	77305.3	{73379., 79744.}	80.6325	0.256537
44	newly 62 weeks cumul	65197.	92080.2	{92396., 93148.}	41.2339	0.14958
45	newly 60 weeks cumul	60722.	88669.	{87758., 89770.}	46.0245	0.164406
46	newly 59 weeks cumul	63639.	80291.1	{79671., 81040.}	26.1665	0.100933
47	newly 55 weeks cumul	54744.	76568.7	{73619., 77962.}	39.8667	0.145627
48	newly 60 weeks cumul	61312.	89010.7	{88470., 89780.}	45.1766	0.161874
49	newly 64 weeks cumul	65680.	86786.4	{75417., 93548.}	32.3151	0.120271
50	newly 63 weeks cumul	60105.	93722.6	{93203., 94155.}	55.9315	0.192931
